# Strain Mediated Adaptation Is Key for Myosin Mechanochemistry: Discovering General Rules for Motor Activity

**DOI:** 10.1371/journal.pcbi.1005035

**Published:** 2016-08-05

**Authors:** Biman Jana, José N. Onuchic

**Affiliations:** 1 Department of Physical Chemistry, Indian Association for the Cultivation of Science, Jadavpur, Kolkata, India; 2 Center for Theoretical Biological Physics, Rice University, Houston, Texas, United States of America; Fudan University, CHINA

## Abstract

A structure-based model of myosin motor is built in the same spirit of our early work for kinesin-1 and Ncd towards physical understanding of its mechanochemical cycle. We find a structural adaptation of the motor head domain in post-powerstroke state that signals faster ADP release from it compared to the same from the motor head in the pre-powerstroke state. For dimeric myosin, an additional forward strain on the trailing head, originating from the postponed powerstroke state of the leading head in the waiting state of myosin, further increases the rate of ADP release. This coordination between the two heads is the essence of the processivity of the cycle. Our model provides a structural description of the powerstroke step of the cycle as an allosteric transition of the converter domain in response to the P_i_ release. Additionally, the variation in structural elements peripheral to catalytic motor domain is the deciding factor behind diverse directionalities of myosin motors (myosin V & VI). Finally, we observe that there are general rules for functional molecular motors across the different families. Allosteric structural adaptation of the catalytic motor head in different nucleotide states is crucial for mechanochemistry. Strain-mediated coordination between motor heads is essential for processivity and the variation of peripheral structural elements is essential for their diverse functionalities.

## Author Summary

Molecular motors are perhaps the most important proteins present in the cell. The importance specifically lies with the fact that these proteins use the chemical energy source (such as ATP) of the cell to generate mechanical work and perform a wide range of functionalities. In this article, we generalize the idea of using structure-based models to explore the mechanochemistry of myosin molecular motors in structural terms. We find that a structural adaptation of the motor head domain in post-powerstroke state signals faster ADP release from the trailing head to maintain its processivity while directionality arises from a careful design of peripheral structural elements. These results along with our earlier results on other motors provide a general rule for motor activity.

## Introduction

Motors belonging to myosin superfamily are associated with a host of important cellular functions, including muscle contraction, cytokinesis, chemotaxis, targeted vesicle and organelle transport [1,2,3,4]. These motors perform mechanical work by producing movement on the actin filament powered by ATP hydrolysis [5,6,7]. While in some cases the myosin motors perform a single cycle of ATP dependent force generation and releases from the actin filament, there are examples where myosin motors perform multiple cycles of stepping on actin prior to its detachment [6,8]. The classic example of such processive motor is myosin V which carries cargo inside the cell towards positive end of the actin filament [8]. Myosin VI, however, carries cargo towards negative end of actin filament [9]. The unidirectional movement of these motors is a result of the coupling between nucleotide dependent conformational changes and actin binding/unbinding cycle [7]. Recent experimental studies have made significant progresses in understanding several features of the mechanochemical cycle of myosin. Kinetic study of single headed myosin has suggested that the rate limiting step in the whole cycle is the ADP release [10]. Kinetic experiments have also probed the weak binding states of myosin V [11]. Trybus et. al. has concluded from their kinetic experiments that monomeric myosin V is processive [12]. Structural investigations of myosin motors in different nucleotide bound state and also in the inactive state have improved our understanding significantly [13,14,15]. Several experiments have explored the head-to-head coordination between two heads of myosin motors at dimer level [16,17]. Recently, kinetic experiments have investigated the asymmetry in the ADP release rate between two heads and discussed about the effect of strain [18,19,20,21]. On the other hand using theoretical and computational techniques, the dynamics of motor processivity, [22] coupling between different parts of the motor domain, [23] transition between different states, [24]flexibility and collective vibrations, [25]coarse-gained modeling of mechanochemical cycle, [26,27] design principles for motility, [28] electrostatic origin of directionality [29] have been explored. Despite significant successes by these theoretical and experimental studies, there is still a need for a detailed structure-based comprehensive understanding of the unidirectional movement of these motors and the allosteric coordination between the heads for the processivity.

Biology appears to have developed principles that are used for the specific needs of each of these motor families. Motor function in general needs allosteric adaptation of their active units to different nucleotide binding states that are mediated by competing interactions between strain interactions in the control element and in the motor binding. These general rules were first observed in our earlier work for the kinesin superfamily. A kinesin model was built based on our protein folding ideas of a strong energetic bias towards the native structure (folding funnel) of the unbound motor in solution complemented by competing interactions arising from nucleotide binding/hydrolysis/release and binding/release from the filament to explore the diverse functionalities in the kinesin superfamily [30,31,32]. Our results suggest a set of underlying principles governing the functional cycle of molecular motors in this family. The structural adaptation of the catalytic motor head in different nucleotide states and binding/release from microtubule is quite similar across the family. The nucleotide dependent allosteric transition of the peripheral structural elements like the neck-linker in kinesin -1 (moves towards positive end on microtubule) and the neck-helix junction in Ncd (moves towards negative end on microtubule) were found to be crucial for their directionality [30,31,32]. Additionally, a strain mediated coordination between the two motor heads in terms of nucleotide binding/release was found to be essential for processivity [27]. In a recent study by Zhang et. al. has dissected the whole mechanochemical cycle of kinesin-1 into three stages, including neck-linker docking, anisotropic translational diffusion of the trailing head and binding of the trailing head to αβ tubulin [33].

In the present work, we show that the same principles apply to the myosin functional cycle. Similarly to the kinesin superfamily, a structure-based model of myosin was built using available structural data that was complemented by adding nucleotide binding/hydrolysis/release and actin binding/release to investigate the structural aspects of the mechanochemical cycle. Using these same principles we could identify the mechanisms governing the functional cycle of the molecular motors in the myosin family. We find a structural adaptation of the trailing head under strain that provides crucial coordination between the two heads of myosin in terms of ADP release. This is essential for processivity. A nucleotide dependent allosteric transition of the converter domain is found to be needed for directionality. The opposite directionality in myosin V and VI can be explained in terms of the different peripheral structural elements. As the model has been built from the available crystal structure, we could provide the structural origin of these important conclusions along with allosteric communications between different parts of myosin in structural terms. By doing so, we are able to determine of the molecular details of governing mechanism of myosin. Indeed, this is one of the main highlights of the current study. Earlier modeling studies did not include this level of structural details. These results for myosin and kinesin strongly suggest that there are general rules/principles that govern the motor functionality across different superfamilies.

## Results and Discussion

### Mechanochemical cycle of a single-head myosin

**Fig 1:**
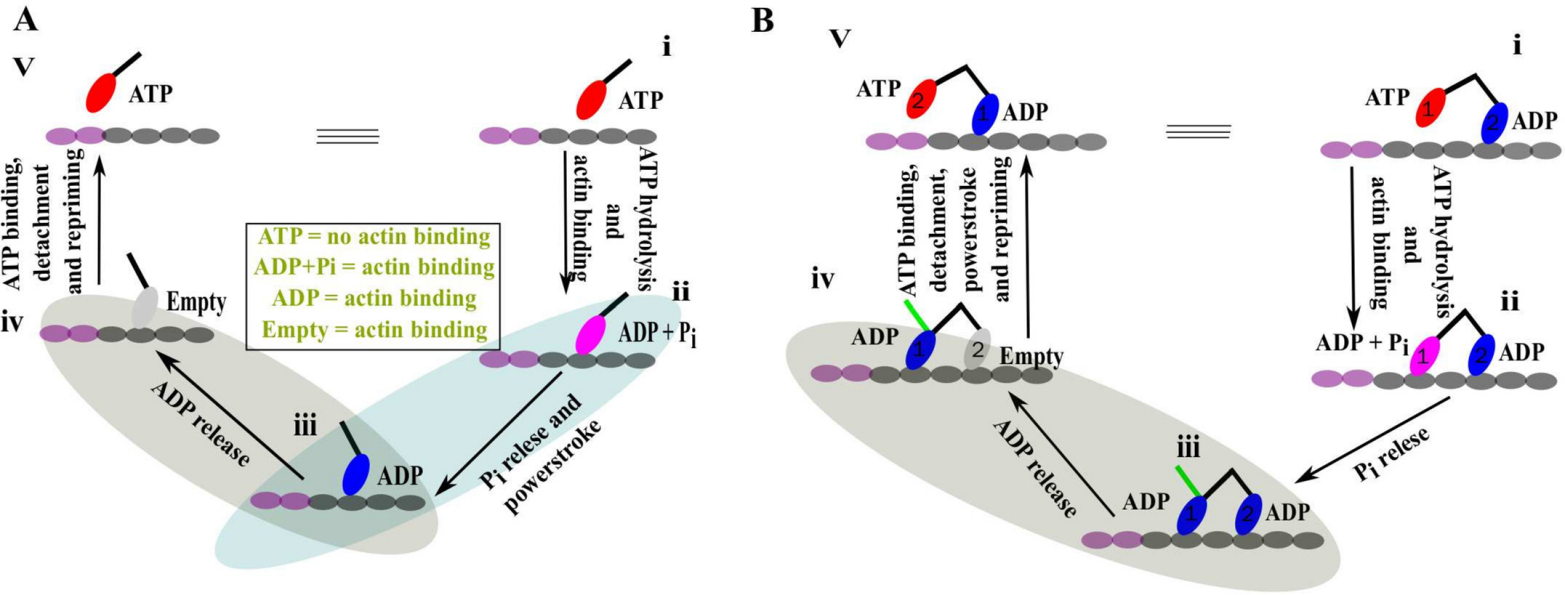
Functional cycle of single- and double-headed myosin. (A) Sequence of events of the mechanochemical cycle of the single-headed myosin. ATP bound (red) head (i) binds to the actin filament followed by the hydrolysis of ATP. The arm (black line on head) of this actin bound head (ii) in ADP + P_i_ state (pink) has a pre-powerstroke conformation. Phosphate (P_i_) release induces the powerstroke conformational change to the lever arm (iii). Next, ADP (blue) is released from the bound head while keeping the post-powerstroke lever arm conformation (iv). Finally, the empty head (gray) detaches from the actin followed by an ATP intake. The ATP dependent unbound head experiences a repriming event to its stable pre-powerstroke lever arm conformation (v). Note that, state (v) is exactly same as state (i) with an additional stepping towards left and also the nucleotide dependent actin binding affinity information in the middle. (B) The sequence of events of the functional cycle of the double-headed myosin. In state (i), head 1 is in an ATP bound state and head 2 is bound to actin in an ADP state. ATP hydrolysis and subsequent binding to actin by head 1 provide a two-head bound myosin (ii). Head 1 releases phosphate (P_i_) to transform into state (iii). In contrary to a single-head myosin, here, after P_i_ release, head 1 cannot perform powerstroke while head 2 is still bound. The green line shows the expected lever arm conformation of head 1. It is important to note here that the two-head-bound state iii could adopt an alternative conformation with the converters of both heads in a post-powerstroke conformation while the lever arm of the leading/trailing head bends backward/forward. Next, ADP releases from head 2 (iv) and subsequently ATP binds to the empty head. Head 2 now detaches from the actin and head 1 now performs its postponed powerstroke step. Finally, head 2 also perform its spontaneous repriming event to form state (v). Note that, state (v) is exactly same as state (i) with an additional stepping towards the left.

Recent experimental and structural data have revealed the different intermediates and the sequence of events of the stepping cycle of myosin motors [5,7,13]. Myosin motors, especially myosin V, can accomplish stepping with a single head (Fig 1A) [12]. The cycle starts with the ATP-bound myosin head (state i) which does not have actin binding ability. This state has a pre-powerstroke lever arm conformation pointing towards the right. After hydrolysis, the head with the ADP and the inorganic phosphate (P_i_) (ADP +Pi state) binds to the actin filament (state ii) while still keeping its pre-powerstroke lever arm conformation. In the next step, the lever arm changes its conformation from the pre to the post-powerstroke conformation (from right-directed to left-directed) simultaneously with the release of the inorganic phosphate (P_i_) while still bound to the actin (state iii). This event is usually termed as powerstroke. Then, the bound head releases ADP to provide an actin bound empty head state (state iv). This empty head, now, binds to ATP and subsequently releases from actin. Once released from the actin, the post-powerstroke conformation of the lever arm, generally, converts to the pre-powerstroke lever arm state (state v). This step is termed repriming event. As it is clearly evident, state v is exactly same as state i with an additional stepping towards the left. Therefore, the cycle can repeat with the same sequence of events. We emphasize the two most important steps of the cycle that are responsible for the directionality as well as the progression of the cycle. In the powerstroke step (ii → iii), the release of P_i_ changes the lever arm conformation of the bound head towards a definite direction (towards positive end of the actin for Myosin V). This step crucially determines the directionality of the motor. In the next step (iii → iv), the ADP release should be faster in this conformation to allow for a faster access for the ATP to bind the head which is important for the speeding up of the cycle.

### Mechanochemical cycle of a double-headed myosin

As described for the single myosin head, the motor detaches from the actin after the completion of each step. On the other hand, the double-headed myosin (myosin V and myosin VI) performs several consecutive cycles of unidirectional movement without being separated from the actin [8,9]. Such a processive movement requires coordination between the two heads for optimal functioning. In Fig 1B, the mechanochemical cycle for the double-headed myosin is presented. It starts when the head 1 is bound to the ATP and head 2 is bound to the ADP (state i). The conformation of the lever arm of the head 1 is in a pre-powerstroke conformation, and it is not bound to actin as in the case of the single-headed myosin. Head 2, on the other hand, is bound to the actin with post-powerstroke lever arm conformation. Next, ATP hydrolysis and subsequent binding of the head 1 while the head 2 is still bound to actin leads to a two-head bound state (state ii). As we show later that this motor takes steps towards the left, the head 1 with ADP + P_i_ is referred as leading head with pre-powerstroke lever arm conformation. The ADP bound head 2, however, is in post-powerstroke lever arm conformation and called as trailing head. The head 1 (leading head) releases P_i_ to provide a state with both heads bound to the ADP and to actin (state iii). As discussed in the earlier section, the ADP bound leading head performs the powerstroke step to achieve its stable post-powerstroke lever arm conformation (the stable lever arm conformation of head 1 is shown in green). However, the powerstroke step is delayed for the head 1 because head 2 (the trailing head) is still bound to the actin. This state is referred as powerstroke-postponed waiting state. Now, the release of ADP from the head 2 (trailing head) while still bound to actin results in state iv. Head 2(trailing head) subsequently binds to the ATP and detaches from the actin. Now, once the head 2 is not bound to actin, head 1 (leading head) performs its waited powerstroke step. The head 2 also performs its repriming event towards the pre-powerstroke lever arm conformation (state v). State v is exactly the same as state i with an additional step towards the left. Therefore, in state v, the identity of the leading and trailing head is interchanged and thus the cycle continues. In addition to the powerstroke step which is important for the directionality, the ADP release step is crucial for the processivity (iii → iv). In state iii, if the leading head (head 1) releases ADP faster than the trailing head (head 2), then the cycle has now a very different fate. The subsequent binding of ATP to the leading head (head 1) releases it from actin and then eventually the trailing head (head 2) also will be released from actin [19]. The resulting state is a completely unbound myosin dimer and thus the processivity is certainly hampered. However, such kind of termination can only happen if the ATP concentration is fairly high compared to its physiological concentration [20].

Therefore, the coordination between the two heads becomes really important in state iii where the ADP release from the head 2 with post-powerstroke lever arm conformation (trailing head) should be much faster than that from the head 1 with pre-powerstroke lever arm conformation (leading head). In addition, the leading head in state iii provides an extra strain (due to its postponed-powerstroke state) on the trailing head that in turn can favor faster ADP release [18–21]. Here, in this article, we present the molecular origin of this important ADP release and powerstroke step using a structure-based modeling.

### Structural comparison of key conformations in the mechanochemical cycle

**Fig 2:**
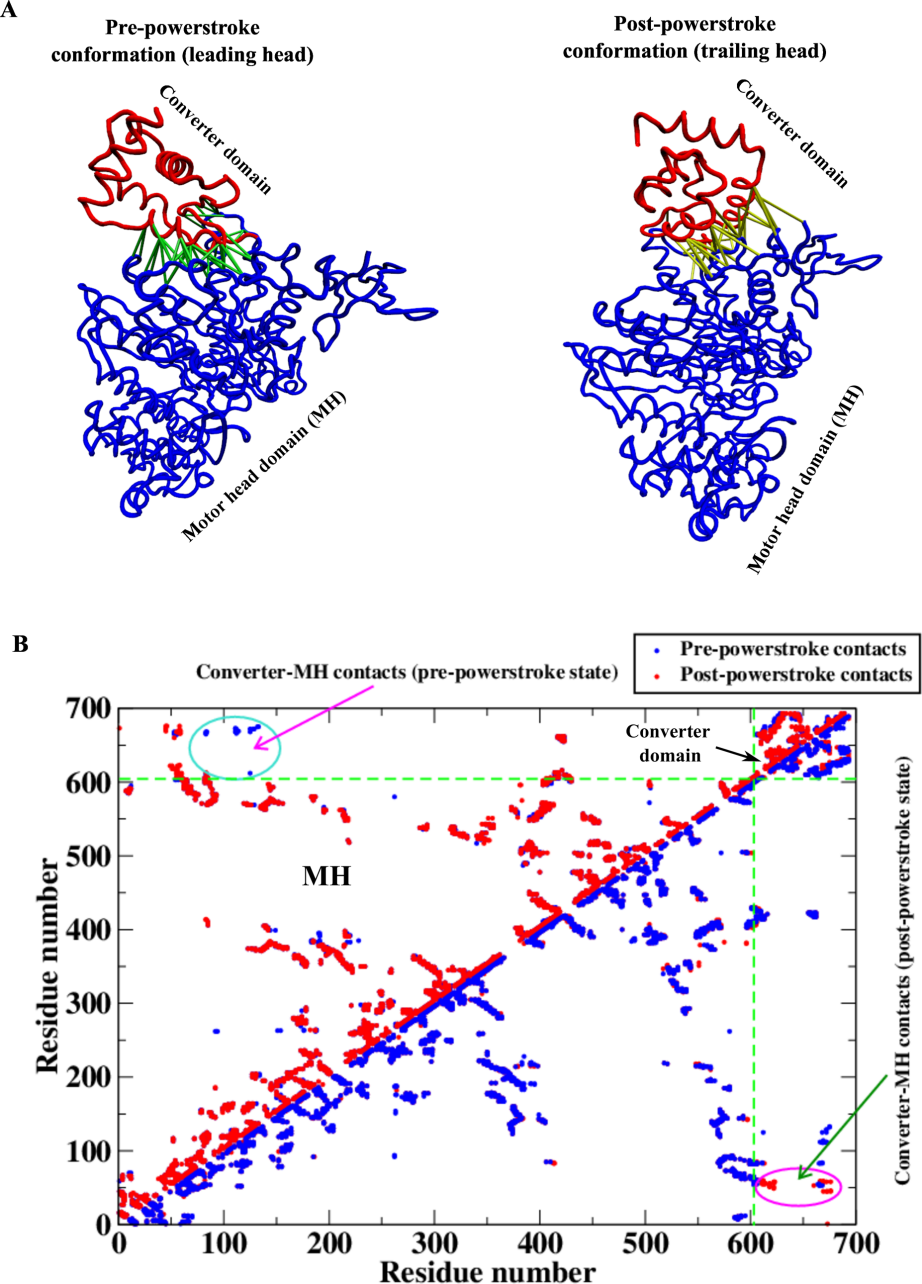
Two important conformational states of the mechanical cycle of myosin. (A) Pre- (PDB 2V26 [34]) and post-powerstroke (PDB 2BHK [36]) states of the myosin motor showing the motor head (MH) and the converter domains. Note the change in the conformation of the converter domain with respect to the MH in the two conformations. The competing interactions (contacts) between the MH and the converter domains, responsible for stabilizing these two states, are shown by lines. (B) Residue-residue contact map for both the conformations. The two dotted green lines are drawn to separate the contact maps of the MH and the converter domains. In the upper triangle, the post-powerstroke contact map (red) is overlaid upon the pre-powerstroke contact map (blue) to identify the exclusive converter-MH contacts in the pre-powerstroke state (shown by lines in Fig 2A). In contrast, the same data have been overlaid in opposite order (blue upon red) in the lower triangle to extract the exclusive converter-MH contacts in the post-powerstroke state (those interactions are shown by lines in Fig 2A).

There are two key conformations of the myosin motor in the mechanochemical cycle described above; the pre-powerstroke (leading head) and the post-powerstroke (trailing head) conformations when bound to actin filament. In the pre-powerstroke state, two conformations have been identified for myosin VI with two nucleotide bound states; ADP.P_i_ and ADP bound [34,35]. While these two conformations have significant changes in terms of actin binding affinity, the converter domain position with respect to motor head domain does not show any significant differences. The structural data of the post-powerstroke conformation is available for myosin VI in the literature [36]. Three-dimensional structures of these two conformations are shown in Fig 2A. Here we show the two important structural parts of myosin motor: (1) the catalytic motor head domain (MH) which binds to the actin filament and nucleotides, (2) the converter domain (that connects to the lever arm) which changes its conformation during the powerstroke to provide the directionality. It is evident from the figure that the major structural difference between these two conformations occurs in the converter domain. While the converter domain points towards the right in the pre-powerstroke conformation, it points towards the left in the post-powerstroke one. It is also worthwhile to note here that both myosins V and VI (although they move towards opposite directions on actin) have similar structural topology for these two conformations. The MH domain of these two motors has overlapping three-dimensional structures. Therefore, understanding the mechanochemistry of one motor is sufficient to extract the overall mechanism governing them. The difference in directionalities comes from differences in the peripheral structural elements that will be discussed later. In Fig 2B, the residue-residue native contact maps of these two conformations are shown. In the upper triangle of this figure, the contact map for the post-powerstroke conformation (red) is pasted upon the pre-powerstroke map (blue). Such a representation reveals the unique pre-powerstroke conformational contacts that are mostly between the converter and MH domains. These contacts are also shown as lines in the pre-powerstroke conformation in Fig 2A. The unique contacts for the post-powerstroke conformations are similarly extracted and are shown as lines in the post-powerstroke conformation. It is important to note that these two sets of contacts describe the essential differences between these two conformations.

### Non-native fluctuation of the MH domain in the post-powerstroke conformation (trailing head) compared to the pre-powerstroke conformation (leading head) in the ADP bound state

**Fig 3:**
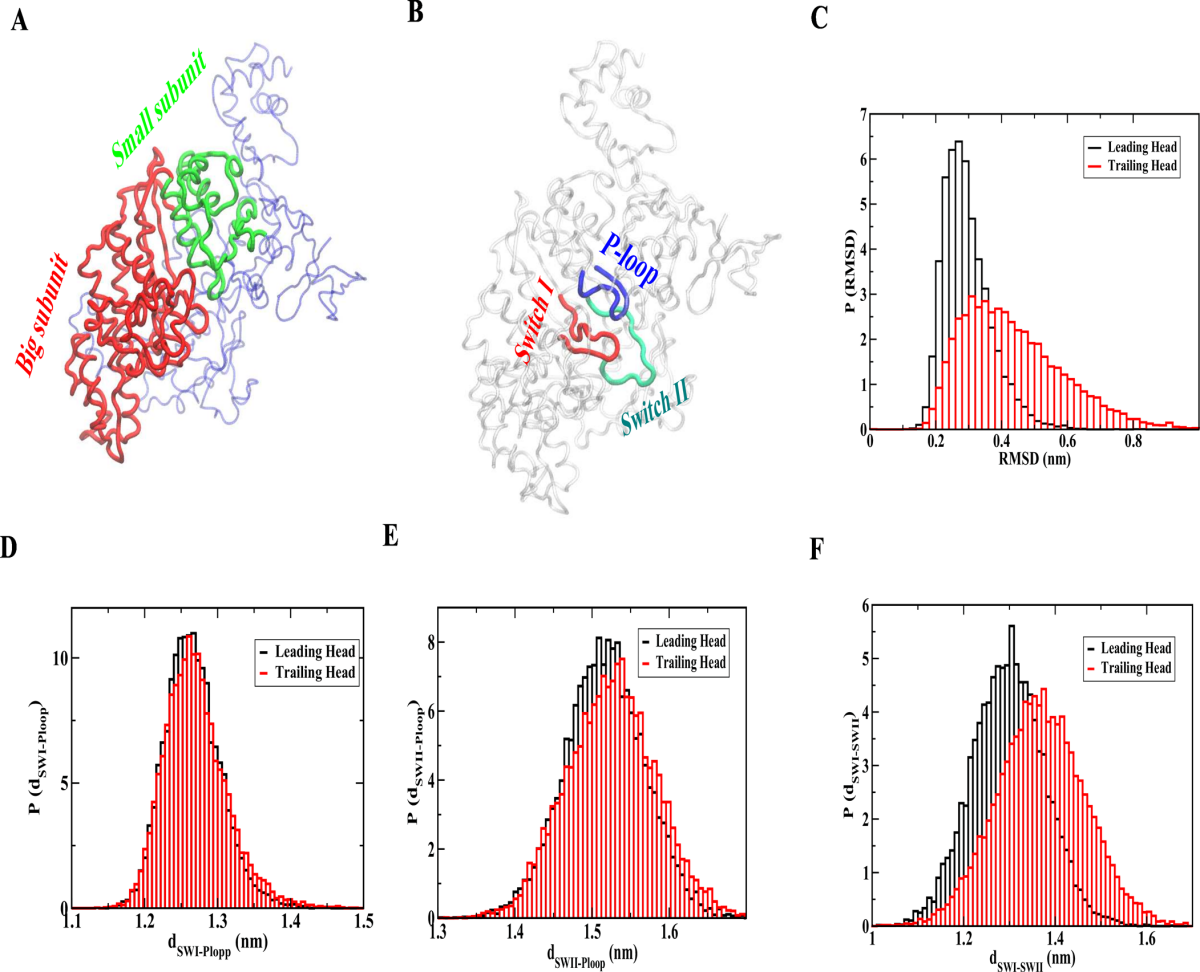
Structural adaptation of the trailing head signals faster ADP release. (A) Myosin MH domain structure showing the big (red) and small (green) subunits. (B) Nucleotide binding region of the MH domain is shown in terms of P-loop (blue), switch I (red) and switch II (green). (C) RMSD distribution (P(RMSD)) of the small subunit of the MH domain after least square fitting of the big subunit from the leading and trailing head simulations. The RMSD is calculated with respect to the pre-powerstroke MH conformation. Note the larger RMSD for the trailing head indicating substantial structural changes. (D) Distribution of distances between P-loop and switch I (P(d_SWI-Ploop_)) for the leading and trailing head simulations. (E) Distribution of distances between P-loop and switch II (P(d_SWII-Ploop_)) for the leading and trailing head simulations. (F) Distribution of distances between switch I and switch II (P(d_SWI-SWII_)) for the leading and trailing head simulations. A larger distance between switch I and switch II for the trailing head simulation compared to leading head signals faster ADP release.

We develop a structure-based model to analyze the non-native fluctuation in the MH domain of the trailing head compared to leading head in the ADP bound state when bound to actin [37,38,39]. In this model, we derive the Hamiltonian for the MH domain based on the pre-powerstroke crystal structure when bound to ADP [35]. The converter domain conformation and the converter-MH contacts of the pre-powerstroke crystal structure are used for the leading head simulation. To investigate non-native structural adaptation of the MH domain of the trailing head, we use the converter domain conformation and the converter-MH contacts of the post-powerstroke crystal structure with MH domain Hamiltonian is kept unchanged. The actin binding interface is extracted from the structural superposition with the actin bound myosin II structure (PDB 1M8Q) [40]. As ADP binds in the interface region between big (red) and small (green) subunit (as shown in Fig 3A) of the MH domain, we first calculate the root mean square deviation (RMSD) of the small subunit after least square fitting of the big subunit relative to the pre-powerstroke (leading head) MH conformation for both the cases. In Fig 3C, the RMSD distributions (P (RMSD)) for the leading and trailing heads are shown. The distribution of the lading head has a peak around 0.28 nm whereas the value for the trailing head is around 0.32 nm with a tail extending up to 0.8 nm. Thus, the change in the conformation of the converter domain has a considerable effect on the structural adaptation of the MH domain. This allosteric adaptation of the trailing head may result in a faster release of ADP that is crucial for the motor processivity (double-headed myosin) or for the speeding up of the detachment from the actin after the powerstroke step (single-head myosin).

### Structural changes near ADP binding domain of the trailing head signals faster release of ADP

In Fig 3B, we show the conserved region of ADP binding pocket in terms of P loop (blue), switch I (red) and switch II (green). We have calculated the distance between those conserved regions from both the simulations to explore specific structural changes. The results are shown in Fig 3D–3F. We find that the distribution of distances between switch I and P loop (P(d_SWI-Ploop_)) does not show any difference when comparing trailing and leading head simulation (Fig 3D). The distribution of distances between switch II and P loop (P(d_SWII-Ploop_)) shifts towards larger distances for the trailing head compared to leading head slightly (Fig 3E). Interestingly, the distribution for the distances between switch I and switch II (P(d_SWI-SWII_)) shows a significant shift towards larger distances for the trailing head compared to leading head simulation (Fig 3E). We have also measured the RMSD of these structural motifs from both the simulations and found a similar conclusion (shown in S1 Fig). Therefore, the nucleotide-binding region of the trailing head adapts a different structure upon the change in the conformation of the converter domain. This might lead to a faster ADP release from the trailing head compared to the leading head. Our result of faster ADP release form the head with post-powerstroke conformation compared to pre-powerstroke conformation is in agreement with previous experimental finding [21]. As discussed earlier, this coordination between the two heads (state iii) is necessary to maintain the processivity. Faster ADP release in turn dictates a faster ATP binding to the trailing head, which after binding to ATP detaches from the actin to maintain the sequence of events correct for optimal functioning. This adaptation to a non-native like structure around the ADP binding pocket in response to a strain (change in the conformation of the converter domain) is commonly referred as cracking [41,42,43].

### Strain on the trailing head helps release ADP faster

**Fig 4:**
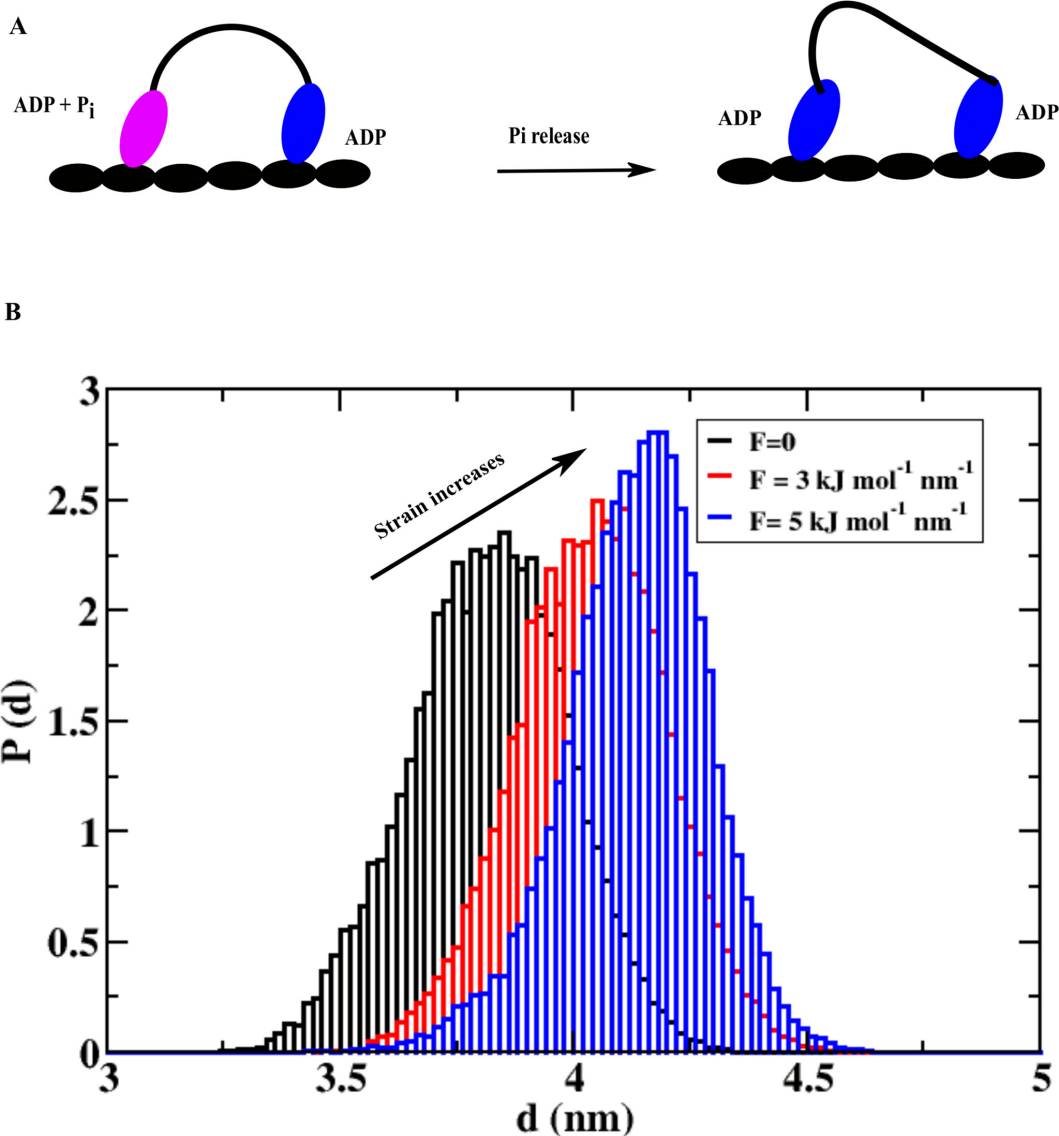
Strain regulates the ADP release kinetics of the trailing head. (A) A schematic representation of myosin dimer showing how P_i_ release can create strain on the trailing head. This strain arises due to the postponed-powerstroke waiting state of the leading head (left head). (B) Probability distribution of distances between small and big subunits of the MH domain at different level of strain on the trailing head. This distribution shifts towards the larger distances as strain increases, which leads to an increase in ADP release rate with increasing strain.

One of the interesting suggestions about the mechanochemical cycle of a double-headed myosin is that the trailing head experiences a forward strain in the waiting state due the postponed-powerstroke nature of the leading head after P_i_ release (state iii in the double-headed cycle) [18–21]. This strain can regulate the faster ADP release of the trailing head which speeds up the whole cycle since ADP release step is rate limiting [10]. In Fig 4A, we show a schematic representation of the strain build up for the trailing head after P_i_ release from the leading head (left head). While the strain should depend on many parameters like strength of the powerstroke, geometry of the waiting region, etc., here we model the strain as the forward pulling force parallel to the actin axis acting on the converter domain. The allosteric response of the strain on the MH domain is calculated by monitoring distance between the small subunit and the big subunit of the MH domain. The distributions of the distances from different simulations with varying forces are shown in Fig 4B. As the strain (force) increases (from 0 to 5 kJ mol^-1^ nm^-1^), we find a gradual shifting of the distance distribution towards larger values.

The peak positions of the distribution are at 3.75 nm, 4.0 nm and 4.2 nm for 0, 3 and 5 kJ mol^-1^ nm^-1^pulling forces, respectively. The larger distance between these two domains at higher strain signals faster ADP release from the trailing head and that essentially speeds up the cycle. One should also note here that F-actin plays a very important role in the mechanochemical cycle of myosin. The above strain on the trailing head is developing because of the fact that the two heads of myosin are tightly bound to the two consecutive binding sites. Without this arrangement or without this binding surface, it is easy to guess that such a strain will not be possible to develop in the waiting state of motor. This is the first computational study on myosin that describes how strain can regulate the speed of the cycle and processivity of myosin.

### Powerstroke mechanism with P_i_ release from the pre-powerstroke conformation

**Fig 5:**
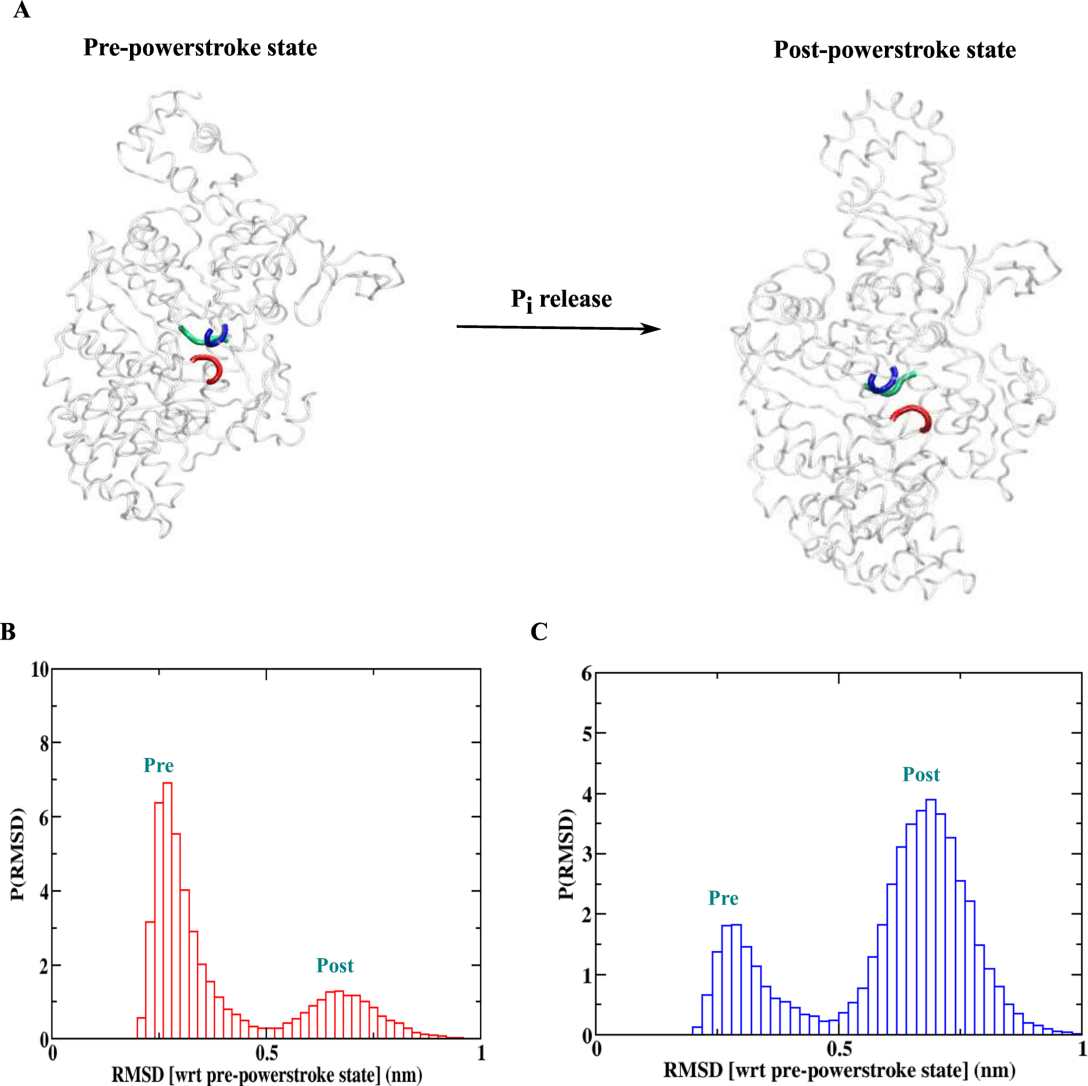
Powerstroke of the myosin motors. (A) Powerstroke step with the release of P_i_. The residues involved in the P_i_ mediated interactions in terms of p-loop (blue), switch I (red) and switch II (green) are shown. (B) RMSD (with respect to pre-powerstroke crystal structure) distribution for the simulation with P_i_ mediated interaction. The population of the pre-powerstroke ensemble is higher. (C) RMSD (with respect to pre-powerstroke crystal structure) distribution for the simulation without P_i_ mediated interactions. Note the inversion of the distribution with phosphate release making post-powerstroke ensemble as the dominant one.

Next, we investigate the powerstroke step that is the central part of this process. During this step, myosin changes its conformation from the pre-powerstroke to the post-powerstroke state. Also the converter domain (that is connected to the lever arm) changes its structure (as shown in Fig 5A), eventually altering the direction of the lever arm. This change in the direction of the lever arm provides directionality to the myosin motor with a displacement of cargo by ~36 nm (for myosin VI) in the direction of its motion. Experiments suggest that the release of P_i_ is important for this step [7]. Here, we build structure-based models with the MH Hamiltonian derived from the pre-powerstroke crystal structure bound to ADP and P_i_ [34].

A dual basin model for the converter domain and converter-MH contacts is derived from the above structure and post-powerstroke structure. In our model, we also keep the interactions between the MH and actin as earlier. Such a model allows the inter-conversion between the two structural states in myosin. In Fig 5A, we also show the important regions in MH domain which are in contact to the P_i_ in terms of the P-loop (blue), switch I (red) and switch II (green). First, we simulate the model including those P_i_ mediated contacts and the results are shown in Fig 5B. The RMSD (calculated with respect to pre-powerstroke crystal structure) probability distribution shows a bimodal behavior. The distribution for the ensemble of the pre-powerstroke conformations is peaked around 0.26 nm and for the post-powerstroke ensemble conformations around 0.7 nm. With the P_i_ mediated contact present in the system, the pre-powerstroke conformations are stable. We then remove the P_i_ mediated contacts from the MH domain. The RMSD probability distribution of the structural ensemble for this simulation is shown in Fig 5C. It is evident that now the post-powerstroke conformation ensemble is largely populated. Therefore, the structural adaptation due to the removal of P_i_ from the MH domain induces the transition from the pre to post powerstroke conformation; this is essentially the powerstroke mechanism. Here, the structural change in the converter region is observed as a consequence of a change occurring in the MH domain. These two structural elements, however, are distant in the three dimensional structure of myosin. This makes this transition as an example of allostery in protein motion. Thus, our simple model captures the essential coordination between the two heads and also explains the powerstroke mechanism in the myosin motors as a consequence of the allosteric structural adaptation. Similar models are used earlier to explore the mechanochemistry of kinesin family of motors (kinesin-1 and Ncd) [30,31,32]. The structural adaptation due to the change in nucleotide states or microtubule binding was the key for their functions. Therefore, it is safe to state that the allosteric structural adaptation due to local perturbation regulates the nucleotide binding/unbinding, filament binding/unbinding or the conformation change of peripheral structural elements. These features are crucial for the function of molecular motors.

### Opposite directionality for myosin V and VI

While myosin V moves towards positive end of the actin, myosin VI moves in the opposite direction. The MH domains of these two motors are structurally very similar with the conserved nucleotide and actin-binding domain. Although, myosin VI has a small insert in the MH domain, it does not influence the directionality [44]. The converter domains for both motors are also structurally very similar. These observations lead to the conclusion that the opposite directionality must be a consequence of the variation of other peripheral structural elements. Indeed, a unique insert present in myosin VI near its lever arm is found to be responsible for directing the lever arm in the opposite direction compared to myosin V [45]. A structural superposition and actin bound state of these two motors are presented in S2 Fig. Therefore, these two motors essentially use the same physical principle for their function with the modification of the peripheral structural elements to accomplish opposite directionality. Indeed, Tsiavaliaris et al have also shown that a forward moving myosin can be engineered to a backward moving myosin by inserting an insert which reveres direction of lever arm extension [46]. We should also mention that a similar strategy of changing the peripheral structural elements to achieve different directionalities is used by the kinesin superfamily of motors (e.g. kinesin-1 and Ncd).

### Concluding remarks

Motor proteins of widely different superfamilies follow the same general working principles. Catalytic motor head domains of different motor proteins of a particular superfamily perform a similar sequence of tasks. The variation of structural elements peripheral to catalytic domains determine different directionalities of their movement. In this work, we reveal that the motors belonging to the myosin family follow general functional rules. The allosteric structural adaptation of the catalytic motor head in response to the different conformations of the converter domain creates asymmetry in the ADP release rates between the two heads. This asymmetry gets enhanced under a forward strain on the trailing head imposed by a postponed powerstroke conformation of the leading head. This coordination between the heads is essential in controlling the processivity of the cycle. An allosteric structural transition of the converter domain is observed upon the P_i_ release from the catalytic head domain and thus determining the structural aspects of powerstroke mechanism. Finally, we explain the diverse functionalities of myosin V and VI (former walks towards positive end and later walks towards negative end of actin) in terms of the variation in structural elements outside of the catalytic domain. These results parallel our earlier results that motors belonging to the kinesin family (kinesin-1 and Ncd) follow the same physical principles to accomplish their mechanochemical cycle and diverse functionalities. Note that all the models have built from the available crystal structure and therefore, we could provide detailed structural aspects of these important conclusions. Additionally, the allosteric communications between different parts of motor proteins are also understood in structural terms. By doing so, we are able to determine the molecular details of governing mechanism of myosin. Earlier modeling studies did not include this level of structural details. This collection of results suggests general underlying principles governing the functionality of molecular motors across different superfamilies.

## Methods

Standard structure-based models implemented in Gromacs (SMOG) were used for the simulations of myosin motors [47]. The structure-based Hamiltonians for different simulations are derived using the crystal structures of two conformations (pre and post powerstroke states) [34,35, 36]. The interface between actin and myosin is extracted from the structural superposition of myosin on the actin bound myosin II structure [40]. Myosin VI motor has an extra insert in the lever arm as compared to Myosin V. To present a general mechanochemical cycle for the myosin motor which can be applied for both Myosin V and Myosin VI, we have not considered the lever arm here.

### The Hamiltonian

The structure of actin bound myosin conformations after structural alignment is used to derive the structure-based model where amino acids are represented by single beads at the location of the C-α atom [37,46,48]. The complete Hamiltonian has the following form:

$$H\left(\left\{{\vec{r}}_{i}\right\}\right)=H_{pre}^{MH}+H_{pre}^{Conv}+H_{post}^{Conv}+H_{pre}^{Conv-MH}+H_{post}^{Conv-MH}+H^{MH-Actin}$$ (1)

#### First term

$H_{pre}^{MH}$ is the Hamiltonian for catalytic motor head in pre-powerstroke state that is given by,

$$\begin{array}{l} H_{pre}^{MH}=H_{pre}^{MH}\left(b\right)+H_{pre}^{MH}\left(nb\right)\\ \;=\sum_{i=1}^{N_{MH}-1}\frac{K_{r}}{2}{\left(r_{i,i+1}-r_{i,i+1}^{0}\right)}^{2}+\sum_{i=1}^{N_{MH}-2}\frac{K_{\theta }}{2}{\left(r_{\theta }-r_{\theta }^{0}\right)}^{2}+\sum_{i=1}^{N_{MH}-3}\sum_{n=1,3}K_{\phi }^{\left(n\right)}\left(1-\cos\left[n\left(\phi_{i}-\phi_{i}^{0}\right)\right]\right)+\\ \;\sum_{i=1}^{N_{MH}-4}\sum_{j=i+3}^{N_{MH}}\varepsilon_{h}\left(5{\left(\frac{r_{ij}^{0}}{r_{ij}}\right)}^{12}-6{\left(\frac{r_{ij}^{0}}{r_{ij}}\right)}^{10}\right)\Delta_{ij}+\varepsilon_{l}{\left(\frac{\sigma }{r_{ij}}\right)}^{12}\left(1-\Delta_{ij}\right) \end{array}$$ (2)

Here, first three terms are the bonded or local part of the Hamiltonian ($H_{pre}^{MH}\left(b\right)$), which consists of harmonic bond constrain between adjacent residue with *K*_*r*_ = 20 kcal/(mol×Å^2^), harmonic angle constrain among three consecutive residue with *K*_*θ*_ = 20 kcal/(mol×rad^2^) and represents the dihedral angle potential with $K_{\phi }^{\left(1\right)}$ = 1.0 kcal/mol and $K_{\phi }^{\left(3\right)}$ = 0.5 kcal/mol that describes the rotation of the backbone involving successive residues from *i* to *i*+3. The fourth term of the Hamiltonian (Eq 2) represents non-bonded interaction potential ($H_{pre}^{MH}\left(nb\right)$). The 10–12 Lennard-Jones potential is used to describe the interactions that stabilize the native contacts. A native contact is defined for a pair of residues (*i* and *j*), if the distance between them is less than 8 Å in the native state and (*i*−*j*)>3. If *i* and *j* residues are in contact in the native state, Δ_*ij*_ = 1; otherwise Δ_*ij*_ = 0. For all non-native pairs for which Δ_*ij*_ = 0, we used repulsive potential with σ = 4Å. We set ε_h_ = ε_l_ = 1.0 kcal/mol. The parameters determining the native topology (Eq 2) $r_{i,i+1}^{0}$, $\theta_{i}^{0}$, $\phi_{i}^{0}$, $r_{ij}^{0}$ and Δ_*ij*_ are determined from the crystal structure of pre-powerstroke state.

#### Second term

The second term in the Eq 1, represent the Hamiltonian for the converter domain in pre-powerstroke state ($H_{pre}^{Conv}$) which is defined as.

$$\begin{array}{l} H_{pre}^{Conv}=H_{pre}^{Conv}\left(b\right)+H_{pre}^{Conv}\left(nb\right)\\ \;=\sum_{i=1}^{N_{Conv}-1}\frac{K_{r}}{2}{\left(r_{i,i+1}-r_{i,i+1}^{0}\right)}^{2}+\sum_{i=1}^{N_{Conv}-2}\frac{K_{\theta }}{2}{\left(r_{\theta }-r_{\theta }^{0}\right)}^{2}+\sum_{i=1}^{N_{Conv}-3}\sum_{n=1,3}K_{\phi }^{\left(n\right)}\left(1-\cos\left[n\left(\phi_{i}-\phi_{i}^{0}\right)\right]\right)+\\ \;\sum_{i=1}^{N_{Conv}-4}\sum_{j=i+3}^{N_{Conv}}\varepsilon_{h}\left(5{\left(\frac{r_{ij}^{0}}{r_{ij}}\right)}^{12}-6{\left(\frac{r_{ij}^{0}}{r_{ij}}\right)}^{10}\right)\Delta_{ij}+\varepsilon_{l}{\left(\frac{\sigma }{r_{ij}}\right)}^{12}\left(1-\Delta_{ij}\right) \end{array}$$ (3)

The Hamiltonian has a very similar structure as in MH Hamiltonian (Eq 2). We use the same values for different parameters (*K*_*r*_, *K*_*θ*_, $K_{\phi }^{\left(1\right)}$, $K_{\phi }^{\left(3\right)}$, *ε*_*h*_, *ε*_*l*_ and *σ*) as in Eq 2. The parameters determining the native topology in Eq 3 ($r_{i,i+1}^{0}$, $\theta_{i}^{0}$, $\phi_{i}^{0}$, $r_{ij}^{0}$ and Δ_*ij*_) are determined from the crystal structure of pre-powerstroke state.

#### Third Term

$H_{post}^{Conv}$ represents the Hamiltonian of converter region in the post-powerstroke state has a similar form to Eq 3 but with the parameters for native topology derived from the post-powerstroke crystal structure.

#### Fourth Term

$H_{pre}^{Conv-MH}$ describes the interaction between MH and converter domain in the pre-powerstroke state. This part of the Hamiltonian is non-bonded in nature and has the following form,

$$H_{pre}^{Conv-MH}=\sum_{i=1}^{N_{Conv}}\sum_{j=1}^{N_{MH}}\varepsilon_{h}\left(5{\left(\frac{r_{ij}^{0}}{r_{ij}}\right)}^{12}-6{\left(\frac{r_{ij}^{0}}{r_{ij}}\right)}^{10}\right)\Delta_{ij}+\varepsilon_{l}{\left(\frac{\sigma }{r_{ij}}\right)}^{12}\left(1-\Delta_{ij}\right)$$ (4)

Parameters in this equation have same values and the native parameters ($r_{ij}^{0}$ and Δ_*ij*_) are extracted from pre-powerstroke structure.

#### Fifth term

$H_{post}^{Conv-MH}$ contains the information about the interaction between MH and converter region in the post-powerstroke state. This term has a similar form to Eq 4. The native parameters ($r_{ij}^{0}$ and Δ_*ij*_) are extracted from post-powerstroke structure.

#### Last term

*H*^*MH*–*Actin*^ represents the interaction between MH and actin that is derived from the aligned structure. This is a non-boned type Hamiltonian with following form,

$$H^{MH-Actin}=\sum_{i=1}^{N_{Actin}}\sum_{j=1}^{N_{MH}}\varepsilon_{h}\left(5{\left(\frac{r_{ij}^{0}}{r_{ij}}\right)}^{12}-6{\left(\frac{r_{ij}^{0}}{r_{ij}}\right)}^{10}\right)\Delta_{ij}+\varepsilon_{l}{\left(\frac{\sigma }{r_{ij}}\right)}^{12}\left(1-\Delta_{ij}\right)$$ (5)

The parameters (*ε*_*h*_, *ε*_*l*_ and *σ*) have the same value as above and the native parameters ($r_{ij}^{0}$ and Δ_*ij*_) are extracted from the aligned structure.

For different simulations, we switch on or off different terms in the Hamiltonian (Eq 1) as described below.

#### Pre- and post-powerstroke state simulation

To simulate the pre-powerstroke state, we switch off the third and fifth terms in Eq 1. For the post-powerstroke state simulation, we switch off second and fourth terms in Eq 1.

#### Powerstroke step simulation

For the simulations to understand powerstroke step mechanism, we did not switch off any term in Eq 1but we introduce the release of inorganic phosphate (P_i_), the phosphate mediated native contacts are removed from the MH Hamiltonian part ($H_{pre}^{MH}$).

### Simulation details

For every simulation, an initial structure is relaxed under the SB Hamiltonian followed by Langevin dynamics simulations at the low-friction limit at T = 300 K to sample the equilibrium structural ensemble (this limit speeds sampling even if friction may be larger in the real system). The equation of motion for the Langevin dynamics used for integration is

$$m{\ddot{\vec{r}}}_{i}=-\zeta {\dot{\vec{r}}}_{i}-\partial_{\vec{r}}H\left(\left\{{\vec{r}}_{i}\right\}\right)+{\vec{\Gamma }}_{i}\left(t\right)$$ (6)

where ζ is the friction coefficient, $-\partial_{\vec{r}}H\left(\left\{{\vec{r}}_{i}\right\}\right)$ is the conformational force. ${\vec{\Gamma }}_{i}\left(t\right)$ is the random force satisfying $\left\langle \vec{\Gamma_{i}}\left(t\right)\cdot \vec{\Gamma_{j}}\left(t'\right)\right\rangle =\left(6\varsigma k_{B}T/h\right)\delta_{ij}\left(t-t'\right)$ where integration time *h* is discretized. In this dynamics we chose ζ = 0.05 $\tau_{L}^{-1}$ and *h* = 0.0025 *τ*_*L*_ with τL=(mσ2/εh)12. Low friction was chosen for the purpose of effective conformational space sampling [49].

## Supporting Information

S1 Fig Non-native structural fluctuation of the ADP binding pocket of the trailing head. (A) Distribution of RMSD of P-loop for the leading and trailing head simulations. (B) Distribution of RMSD of switch I for the leading and trailing head simulations. (C) Distribution of RMSD of switch II for the leading and trailing head simulations. All the RMSDs are calculated with respect to the pre-powerstroke crystal structure conformation. A larger RMSD for switch I and switch II for the trailing head simulation compared to leading head indicates significant non-native fluctuations and in turn signals faster ADP release.
(EPS)

S2 Fig Peripheral structural element determines directionality in myosin V & VI. (A) The post-powerstroke conformations of myosin V and myosin VI including the lever arms. Note the extra insert between the converter domain and lever arm for myosin VI that is responsible for the opposite directions for the lever arms in the different myosins. A structural overlap of these two motors shows that while the MH domains of both the motors are very similar, the extra insert provides different directionality to the lever arms. (B) Myosin V and VI are overlapped on the actin bound myosin II structure to obtain actin bound conformation for both motors. Note the orientation of lever arm for myosin V (positive end) and myosin VI (negative end).
(EPS)
